# Supplementary material for: Antimicrobial resistance and virulence analysis of Escherichia coli carried by black-faced spoonbill (Platalea minor) in Liaoning, China
Source: PLoS One. 2026 Jan 2;21(1):e0339376. doi: 10.1371/journal.pone.0339376 (PMC12758694; doi:10.1371/journal.pone.0339376)
Supplement: S1 File — Table S1. Based on CLSI, the minimum inhibitory concentration (MIC) of 12 antibiotics for Enterobacteriaceae is shown with the concentration range of the E-test. Table S2. Primers for detecting virulence genes used in this study. Table S3. Accession numbers of the BioProject and BioSample for the genomes of 16 E. coli isolates. (DOCX) [file pone.0339376.s001.docx]

# Supporting information

**Table S1 Based on CLSI, the minimum inhibitory concentration (MIC) of 12 antibiotics for Enterobacteriaceae is shown with the concentration range of the E-test.**

| Antibiotic | | MIC Interpretive Criteria  (µg/mL) | | | MIC range tested (µg/mL) |
| --- | --- | --- | --- | --- | --- |
|  |  | S | I | R |  |
| Tetracyclines | Tetracycline | ≤4 | 8 | ≥16 | 0.016-256 |
| Penicillins | Piperacillin | ≤16 | 32-64 | ≥128 | 0.016 - 256 |
|  | Ampicillin | ≤8 | 16 | ≥32 | 0.016-256 |
| Penicillins/β- Lactam enzyme inhibitor complex | Ampicillin- Sulbactam | ≤8/4 | 8/16 | ≥32/16 | 0.016 - 256* |
| Sulfonamides | Trimethoprim-Sulfamethoxazole | ≤2/38 | - | ≥4/76 | 0.002-32 |
| First and second generation cephalosporins | Cefazolin | ≤16 | - | ≥32 | 0.016 - 256 |
| Third and fourth generation cephalosporins | Cefotaxime | ≤1 | 2 | ≥4 | 0.002 - 32 |
| Aminoglycosides | Gentamicin | ≤4 | 8 | ≥16 | 0.064 - 1024 |
| Quinolones | Ciprofloxacin | ≤0.25 | 0.5 | ≥1 | 0.002-32 |
|  | Levofloxacin | ≤0.5 | 1 | ≥2 | 0.002 - 32 |
| Chloramphenicols | Chloramphenicol | ≤8 | 16 | ≥32 | 0.016-256 |
| Polypeptide | Colistin | - | ≤2 | ≥4 | - |

**Table S2 Primers for detecting virulence genes used in this study.**

| *E. coli*  pathotype | Gene | Sequence (5=–3=) | Amplicon  size (bp) | reference |
| --- | --- | --- | --- | --- |
| STEC | *Stx1* | F:ATAAATCGCCATTCGTTGACTAC | 180 | [4] |
|  |  | R:AGAACGCCCACTGAGATCATC |  |  |
|  | *Stx2* | F:GGCACTGTCTGAAACTGCTCC | 255 | [4] |
|  |  | R:TCGCCAGTTATCTGACATTCTG |  |  |
| EPEC/STEC | *eae* | F:TCAATGCAGTTCCGTTATCAGTT | 482 | [4] |
|  |  | R:GTAAAGTCCGTTACCCCAACCTG |  |  |
| ETEC | *LT* | F:ATTTACGGCGTTACTATCCTC | 281 | [4] |
|  |  | R:TTTTGGTCTCGGTCAGATATG |  |  |
|  | *STa* | F:GCTAATGTTGGCAATTTTTATTTCTGTA | 190 | [4] |
|  |  | R:AGGATTACAACAAAGTTCACAGCAGTAA |  |  |
|  | *STb* | F:GCCTATGCATCTACACAATC | 279 | [4] |
|  |  | R:TGAGAAATCGACAATGTCCG |  |  |
|  | *ST* | F:TCTGTATTGTCTTTTTCACCTTTC | 165 | [4] |
|  |  | R:TTAATAGCACCCGGTACAAGC |  |  |
| EAEC | *EAST-1* | F:CCATCAACACAGTATATCCGA | 111 | [4] |
|  |  | R:GGTCGCGAGTGACGGCTTTGT |  |  |

**Table S3 Accession numbers of the BioProject and BioSample for the genomes of 16 *E. coli* isolates**

| **Strain name** | **Bioproject_accession** | **Biosample_accession** |
| --- | --- | --- |
| 883 | PRJNA977261 | SAMN35523939 |
| 890 | PRJNA977423 | SAMN35523940 |
| 937 | PRJNA977425 | SAMN35523941 |
| 1018 | PRJNA977426 | SAMN35523942 |
| 822red | PRJNA977428 | SAMN35523943 |
| 826red | PRJNA977429 | SAMN35523944 |
| 830red | PRJNA977430 | SAMN35523945 |
| 837red | PRJNA977431 | SAMN35523946 |
| 848red | PRJNA977433 | SAMN35523947 |
| 887 | PRJNA977434 | SAMN35523948 |
| 892 | PRJNA977435 | SAMN35523949 |
| 926 | PRJNA977436 | SAMN35523950 |
| 958 | PRJNA977437 | SAMN35523951 |
| 970 | PRJNA977438 | SAMN35523952 |
| 984 | PRJNA977439 | SAMN35523953 |
| 994 | PRJNA977440 | SAMN35523954 |
