# Supplementary material for: Antimicrobial resistance and virulence analysis of Escherichia coli carried by black-faced spoonbill (Platalea minor) in Liaoning, China
Source: PLoS One. 2026 Jan 2;21(1):e0339376. doi: 10.1371/journal.pone.0339376 (PMC12758694; doi:10.1371/journal.pone.0339376)
Supplement: Attachment — Submitted filename: MMN PONE-D-23-42911.pdf [file pone.0339376.s002.pdf]

PONE-D-23-42911 – Reviewer: MMN

In general, this paper is interesting, and the authors have made an obvious effort. However, as a reviewer, I find this version of the paper requires more efforts to be valid to gain the attention of other researchers and readers. I have listed some points that could help in that manner.

Manuscript in general requires English writing enhancement

Abstract

Line 20: This sentence is not clear:
"Seventy-four samples has *E. coli* isolated (29.7%), 43.2% of isolates carried an antimicrobial resistant phenotype and 21.6% isolates were considered to be MDR strains."

Lines 30-35: This paragraph needs to be rewritten in a way that would highlight the importance and relevance of their findings to public health and to the reader's knowledge:

"In summary, *E. coli* may serve as a reservoir of resistance and virulence genes in migratory birds and may be transmitted to other species during migration, with virulent or multidrug resistant *E. coli* a potential threat to the rare black-faced spoonbill and to human public health".

Introduction

The introduction should describe the main pathogen first (*E. coli*) and its importance as a main reservoir for transmission of ARGs to other pathogens. Then, a short but informative few paragraphs about migratory birds and black-faced spoonbills would provide a strong introduction to the proposed role of black-faced spoonbills in spreading pathogens and antibiotic-associated genes.

Materials and methods

Over all, the authors clearly put in a lot of efforts to finalise this paper. However, crucial questions have been raised about their methodologies.

1: Using boiling method for DNA extraction:

For some researchers, it is accepted once the boiling method is used for bacterial DNA extraction. It would provide a low-cost method. However, it may provide low-quality DNA that may affect the results of the detection of virulence and drug-resistant associated genes.

2: Using an automated microbial and antimicrobial identification system

It is well known (the authors have also stated that clearly in their discussion) that the commercially available automated microbial and antimicrobial identification systems are providing results with

variable and inconsistent accuracies. Therefore, authors should describe the obtained accuracy rate following using that system for identification of *E. coli*.

3: The authors decided to use conventional PCR assay to detect virulence and antibiotic resistant associated genes in the first place, then they moved to use SGS assay to screen only 16 isolates under just one term which is the MDR phenotype.

This provides an impression about that the authors might not be quite sure about the primary results of *E. coli* identification and MDR phenotypes. Therefore, it would be a good suggestion if the authors would change the story of this paper to describe the conventional methods at first and then describe the addition of the SGS assay to unravelling the mysteries behind some inconsistent results.

4: Why have the authors were decided to use SGS over NGS or WGS? The SGS system has some issues with sequence depths that may affect the perfect annotations and sequence closing.

Results

1: Visualise the phylogenetic tree of the 16 isolates with adding the host and sample location to each isolate was very nice and informative. It is a good choice from the authors.

2: It seems that isolates number 937 and 970 were the only two significant isolates that have been chosen to be sequenced so far. They share significant similarity and close relationship with two other isolates from cattle and Mink, respectively. The authors are encouraged to pay more effort to generate genome comparison between isolate 937 and LNF11 and between isolate 970 and 112 as well. This would make the story more interesting.

Discussion

1: The discussion clearly showed that the authors were not biased about their methodology and findings.

2: As mentioned earlier (section 3 – Material and Methods), the discussion section needs to be re-written in a more confident way about the results. The current version of discussion provides an impression of uncertainty about the results.

3: In line 332, the authors have mentioned that strain 112 was isolated from Liaoning cows but in the figure of phylogenetic tree it was noted as isolated from Mink. They need to correct that using the proper source please.

4: In line 336: The authors mentioned some relationship between strains 958 and CX144T due to their localisation in a same branch. The phylogenetic tree does not imply this statement.
